# Supplementary material for: Using regulatory variants to detect gene–gene interactions identifies networks of genes linked to cell immortalisation
Source: Nat Commun. 2020 Jan 17;11:343. doi: 10.1038/s41467-019-13762-6 (PMC6969137; doi:10.1038/s41467-019-13762-6)
Supplement: Supplementary file 1 — Supplementary Information [file 41467_2019_13762_MOESM1_ESM.pdf]

Using regulatory variants to detect gene-gene interactions identifies networks of genes linked to cell immortalisation

Wragg, D., *et al*.

Supplementary Table 1. Summary of gene-gene interactions that can be explained in LBC1936 and GEUVADIS datasets

	LBC	GEUVADIS	BOTH
Number of genes with significant PrediXcan models	1205		311
Number of significant veQTL	129	201	87
Number of significant GxG interactions	237291		3953
Number of significant concordant GxG interactions			2821
GxG interactions explained by nearby varaint	12	7	
GxG interactions explained by additional gene	271 (2)*	109 (5)*	0
GxG interactions explained by cis-genetic effects only	28	1	1
GxG interactions explained by gene B cis-genetic component	35	50	5
Number of interactions remaining unexplained			2472

* Number in brackets indicates number also identified as being explained by nearby variant

Supplementary Table 2. Gene enrichment analysis across the largest networks. Analysis of enrichment for particular biological pathways and motifs was performed using FUMA among the set of interacting gene[B]s of each gene[A]. The background gene list was all gene[B]s tested (irrespective of significance). The combined set of all non-redundant interacting gene[B]s were also tested in the same way (labelled ALL).

Gene set	Curated gene sets	GO biological process	Hallmark gene sets	TF targets
ALL	DODD NASOPHARYNGEAL CARCINOMA DN; PILON KLF1 TARGETS DN; NUYTTEN EZH2 TARGETS DN; FISCHER DREAM TARGETS; WEI MYCN TARGETS WITH E BOX; PUJANA BRCA1 PCC NETWORK; MARTENS TRETINOIN RESPONSE DN; DIAZ CHRONIC MEYLOGENOUS LEUKEMIA UP; GOBERT OLIGODENDROCYTE DIFFERENTIATION UP; GRAESSMANN APOPTOSIS BY DOXORUBICIN DN	MITOCHONDRION ORGANIZATION; CHROMOSOME ORGANIZATION; PROTEIN LOCALIZATION; CELL CYCLE; CELLULAR RESPONSE TO STRESS; PHOSPHATE CONTAINING COMPOUND METABOLIC PROCESS; DNA METABOLIC PROCESS; RNA PROCESSING; CELL CYCLE PROCESS; CELLULAR RESPONSE TO DNA DAMAGE STIMULUS	INTERFERON GAMMA RESPONSE; INTERFERON ALPHA RESPONSE; E2F TARGETS; G2M CHECKPOINT; MYC TARGETS V1; OXIDATIVE PHOSPHORYLATION; MITOTIC SPINDLE; IL2 STAT5 SIGNALING	SCGGAAGY ELK1 02; GGGCGGR SP1 Q6; RCGCANGCGY NRF1 Q6; GCCATNTTG YY1 Q6; CTTTGT LEF1 Q2
ACCS	WEI MYCN TARGETS WITH E BOX; KINSEY TARGETS OF EWSR1 FLII FUSION UP; PUJANA BRCA1 PCC NETWORK; RODRIGUES THYROID CARCINOMA POORLY DIFFERENTIATED UP; ZHANG BREAST CANCER PROGENITORS UP; FISCHER DREAM TARGETS; PILON KLF1 TARGETS DN; DIAZ CHRONIC MEYLOGENOUS LEUKEMIA UP; PUJANA CHEK2 PCC NETWORK; LEE BMP2 TARGETS DN	MITOCHONDRION ORGANIZATION	G2M CHECKPOINT; MYC TARGETS V1	NA
C17orf97	WEI MYCN TARGETS WITH E BOX; MANALO HYPOXIA DN; KRIGE RESPONSE TO TOSEDOSTAT 24HR DN; FISCHER DREAM TARGETS; GOBERT OLIGODENDROCYTE DIFFERENTIATION UP; SHEDDEN LUNG CANCER POOR SURVIVAL A6; TARTE PLASMA CELL VS PLASMABLAST DN; KRIGE RESPONSE TO TOSEDOSTAT 6HR DN; CAIRO HEPATOBLASTOMA CLASSES UP; DODD NASOPHARYNGEAL CARCINOMA DN	NA	NA	NA
FLVCR1-AS1	GABRIELY MIR21 TARGETS; PILON KLF1 TARGETS DN	NA	NA	NA
LDHC	WONG MITOCHONDRIA GENE MODULE; MOOTHA MITOCHONDRIA; TIEN INTESTINE PROBIOTICS 24HR UP; STARK PREFRONTAL CORTEX 22Q11 DELETION DN; YAO TEMPORAL RESPONSE TO PROGESTERONE CLUSTER 13; MOOTHA HUMAN MITODB 6 2002; WEI MYCN TARGETS WITH E BOX	MITOCHONDRION ORGANIZATION; TRANSLATIONAL TERMINATION; CELLULAR PROTEIN COMPLEX DISASSEMBLY; MITOCHONDRIAL TRANSLATION; TRANSLATIONAL ELONGATION; ORGANONITROGEN COMPOUND METABOLIC PROCESS; PROTEIN COMPLEX SUBUNIT ORGANIZATION;	OXIDATIVE PHOSPHORYLATION	NA

Gene set	Curated gene sets	GO biological process	Hallmark gene sets	TF targets
		SINGLE ORGANISM BIOSYNTHETIC PROCESS; CELLULAR MACROMOLECULAR COMPLEX ASSEMBLY; NUCLEOSIDE TRIPHOSPHATE METABOLIC PROCESS		
OGDHL	FISCHER DREAM TARGETS; GOBERT OLIGODENDROCYTE DIFFERENTIATION UP; DODD NASOPHARYNGEAL CARCINOMA DN; SOTIRIOU BREAST CANCER GRADE 1 VS 3 UP; KOBAYASHI EGFR SIGNALING 24HR DN; KINSEY TARGETS OF EWSR1 FLII FUSION UP; FISCHER G2 M CELL CYCLE; BENPORATH PROLIFERATION; CHIANG LIVER CANCER SUBCLASS PROLIFERATION UP; MARSON BOUND BY E2F4 UNSTIMULATED	CELL CYCLE; MITOTIC CELL CYCLE; CELL CYCLE PROCESS; ORGANELLE FISSION; MITOTIC NUCLEAR DIVISION; CHROMOSOME SEGREGATION; CELL DIVISION; CHROMOSOME ORGANIZATION; SISTER CHROMATID SEGREGATION; NUCLEAR CHROMOSOME SEGREGATION	E2F TARGETS; G2M CHECKPOINT; MITOTIC SPINDLE	NA
SLFN5	MOSERLE IFNA RESPONSE; TAKEDA TARGETS OF NUP98 HOXA9 FUSION 3D UP; HECKER IFNB1 TARGETS; BOSCO INTERFERON INDUCED ANTIVIRAL MODULE; FULCHER INFLAMMATORY RESPONSE LECTIN VS LPS DN; DAUER STAT3 TARGETS DN; TAKEDA TARGETS OF NUP98 HOXA9 FUSION 10D UP; TAKEDA TARGETS OF NUP98 HOXA9 FUSION 16D UP; BROWNE INTERFERON RESPONSIVE GENES; FARMER BREAST CANCER CLUSTER 1	DEFENSE RESPONSE TO VIRUS; RESPONSE TO VIRUS	INTERFERON GAMMA RESPONSE; INTERFERON ALPHA RESPONSE	NA
SYNGR1	CHEN METABOLIC SYNDROME NETWORK; MEISSNER BRAIN HCP WITH H3K4ME3 AND H3K27ME3; JOHNSTONE PARVB TARGETS 3 UP; LIU PROSTATE CANCER DN; BENPORATH ES WITH H3K27ME3	REGULATION OF ERK1 AND ERK2 CASCADE; CELL DEVELOPMENT; NEUROGENESIS; NEGATIVE REGULATION OF DEVELOPMENTAL PROCESS; NEGATIVE REGULATION OF CELL DIFFERENTIATION; REGULATION OF MULTICELLULAR ORGANISMAL DEVELOPMENT; NEGATIVE REGULATION OF MULTICELLULAR ORGANISMAL PROCESS; REGULATION OF MAPK CASCADE	NA	GGGAGGRR MAZ Q6

Supplementary Table 3. Summary of different data pre-processing outcomes on number of gene[A] retained

	None[1]	LMER[2]	LMER+PEER[3]	Pred(LMER_PEER) Obs (LMER)
Significant prediction models in LBC1936	6971	7082	9316	9316
(of which fail kurtosis)	880	1029	578	4283
Probe models with good R2 values	832	922	1960	1387
(representing unique genes)	744	823	1709	1205
Probes with good R2 in GEUVADIS data	360	391	630	444
(representing unique genes)	316	341	534	361
(which fail kurtosis in GEUVADIS data)	23	20	5	366
Number of unique genes replicated by both datasets	258	280	449	311
(of which missing compared to results of no-processing)	-	3	0	36
Significant probe-pair interactions in LBC1936	227231	223259	7158	237291
(of which replicated in GEUVADIS data)	740	333	27	7052
(of which have a consistent direction of effect)	472	225	17	5185

[1] No further processing of normalised observed expression data to address covariates (sex and age) or technical variation (sample plate and Sentrix assay) is performed prior to running PredictDB

[2] Mixed model applied prior to running PredictDB to regress-out covariates (sex and age) and technical variation (sample plate and Sentrix assay fitted as random effects)

[3] In addition to LMER, the data is also processed using PEER to account for hidden covariates prior to running PredictDB

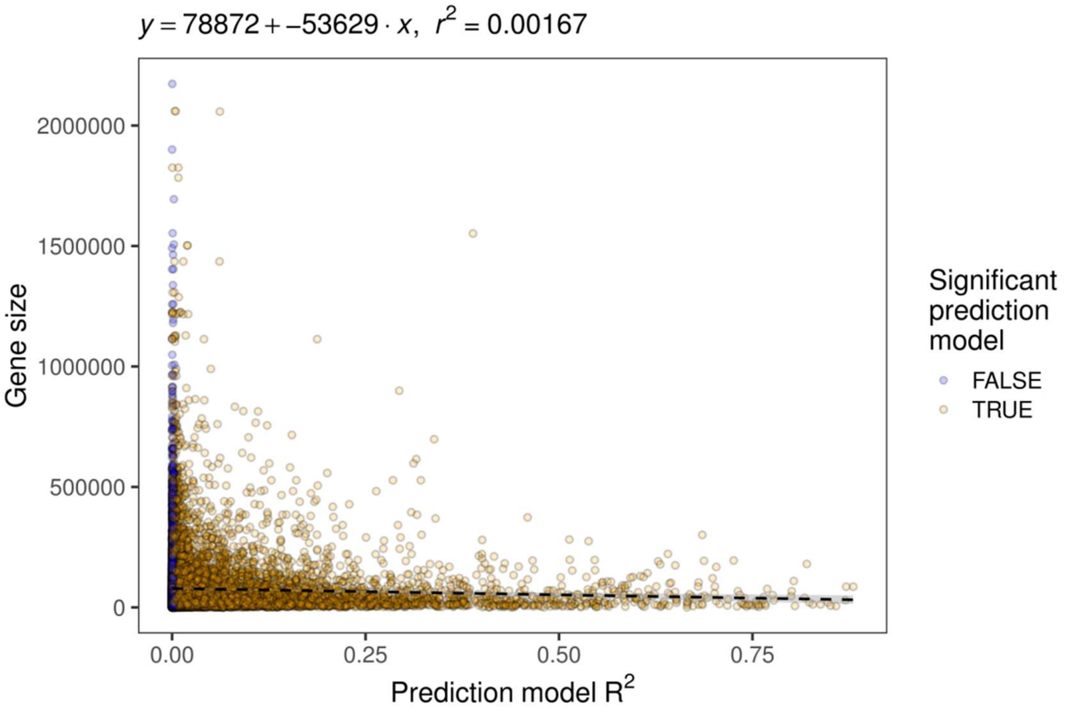

Supplementary Figure 1. Plot of gene size versus R^2 of gene expression prediction model.

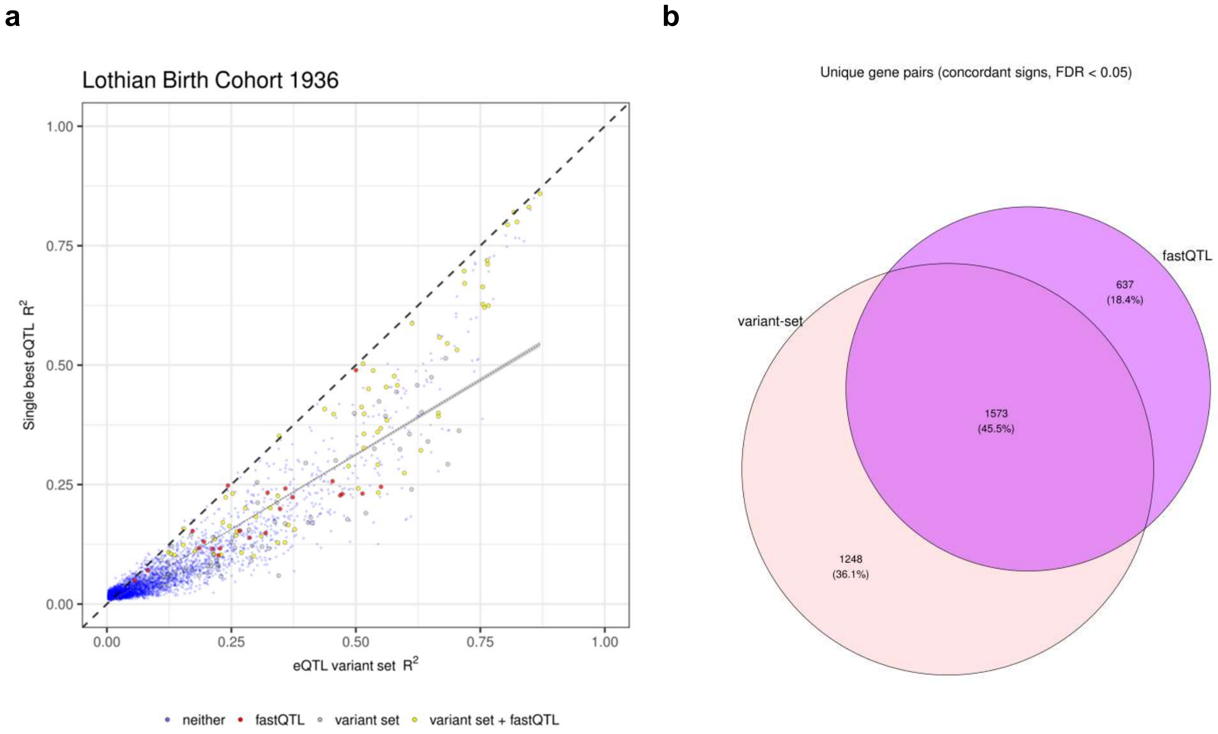

Supplementary Figure 2. Comparison of single best eQTL and variant set approaches.

(**a**) Predicted versus observed R^2 of the single best eQTL model identified by fastQTL, and the eQTL variant set models, illustrates the variant set approach improves predictions. Comparison of significant results from the interaction analyses (**b**) indicates that while a greater number of interactions are identified by the variant set approach, a portion (18.4%) are identified only when using the single best eQTL models, suggesting that the use of multiple *cis*-regulatory variants can potentially sometimes lead to masking effects.

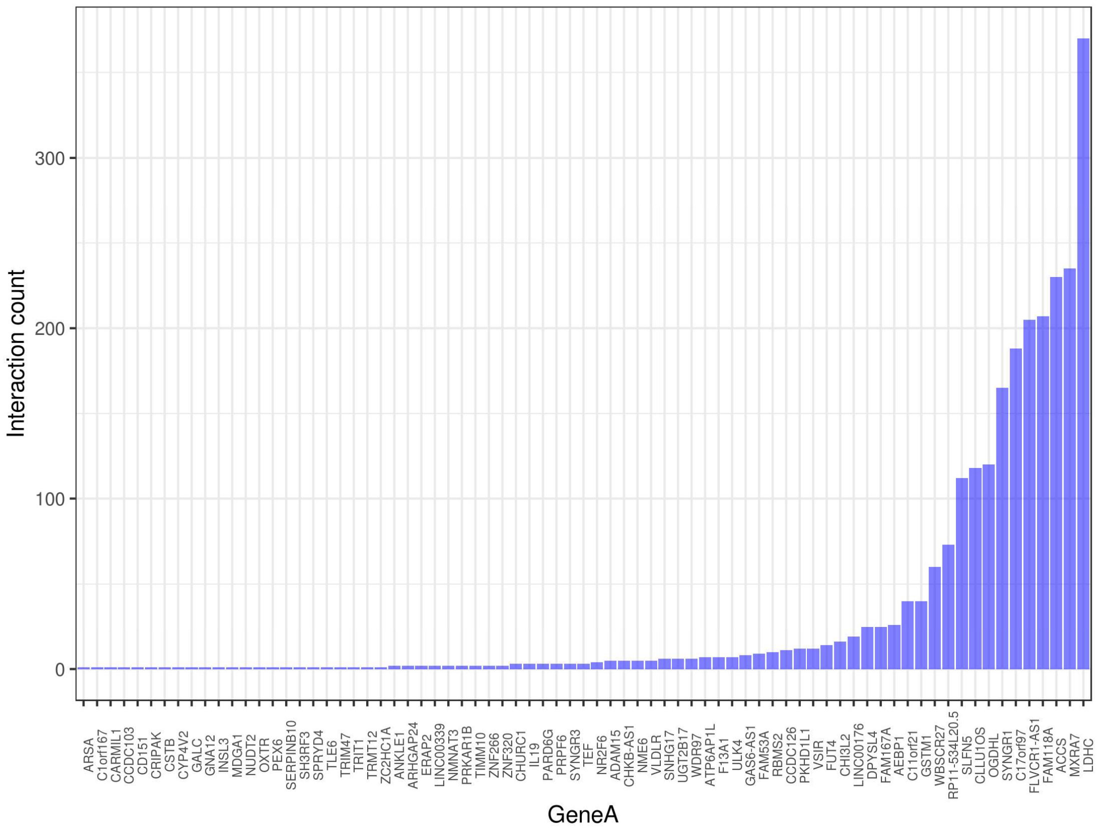

Supplementary Figure 3. Histogram indicating the number of interacting genes identified for each gene[A].

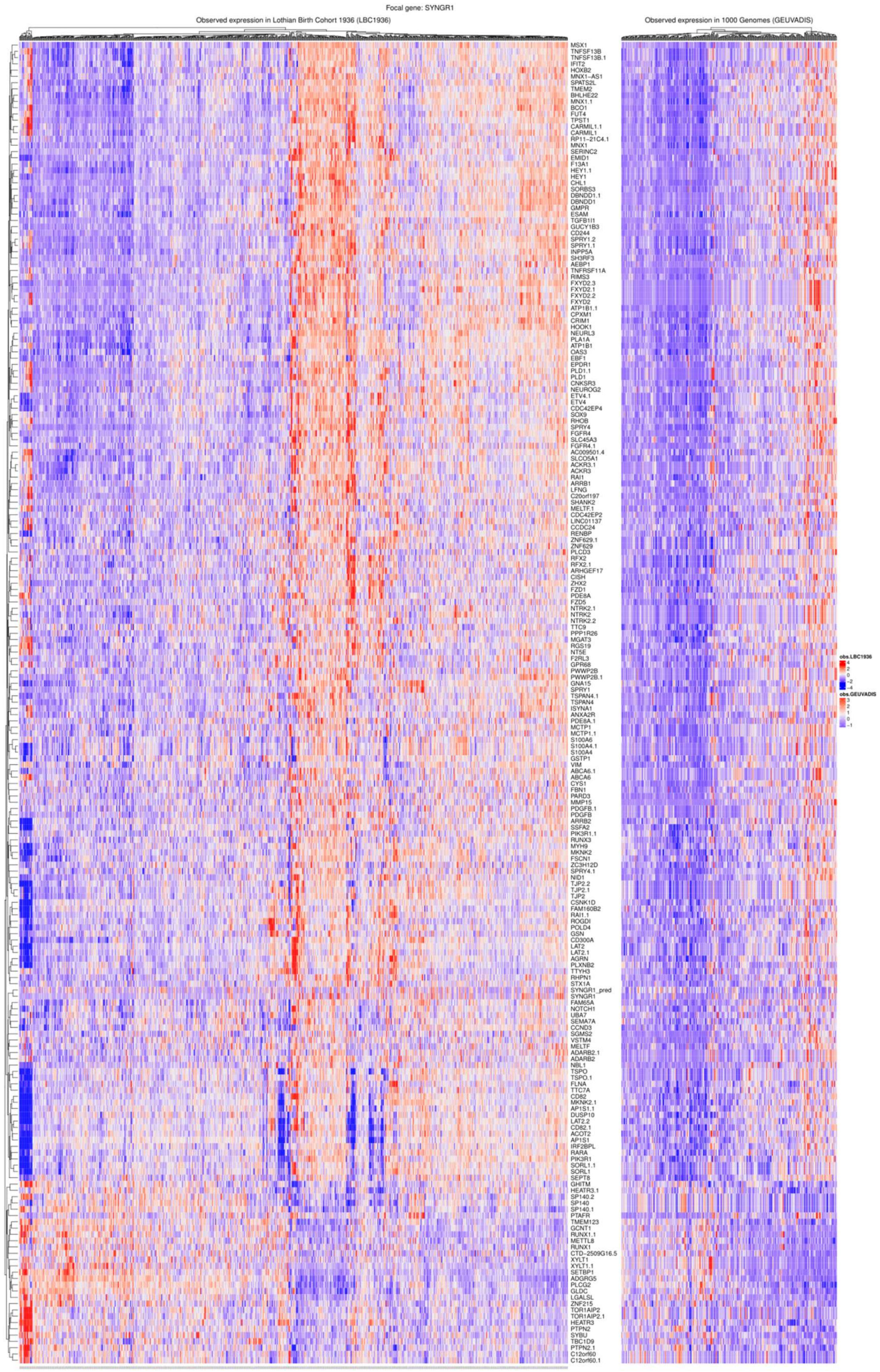

Supplementary Figure 4. The *SYNGR1* gene expression heatmap

Gene expression heatmap for genes that exhibit significant (ANOVA F-test FDR < 0.05) interactions with *SYNGR1* in the Lothian Birth Cohort 1936 and 1000 Genomes datasets. *SYNGR1_pred* is the predicted expression for *SYNGR1*.

a

LBC1936, OXTR:BHLHE22

FDR $(pred^A : obs^B) = 0.0249$

	Estimate	Std. Error	t value	Pr(>|t|)
(Intercept)	−0.648	0.04	−16.2	6.49e−52
predA	0.388	0.0231	16.8	3.04e−55
obsB	0.161	0.0404	4	6.85e−05
predA:obsB	0.0819	0.0233	3.51	0.000474

GEUVADIS, OXTR:BHLHE22

FDR $(pred^A : obs^B) = 0.0112$

	Estimate	Std. Error	t value	Pr(>|t|)
(Intercept)	−3.06	0.422	−7.25	2.78e−12
predA	1.9	0.253	7.52	4.88e−13
obsB	−0.000158	0.0441	−0.00359	0.997
predA:obsB	0.108	0.0259	4.16	4.07e−05

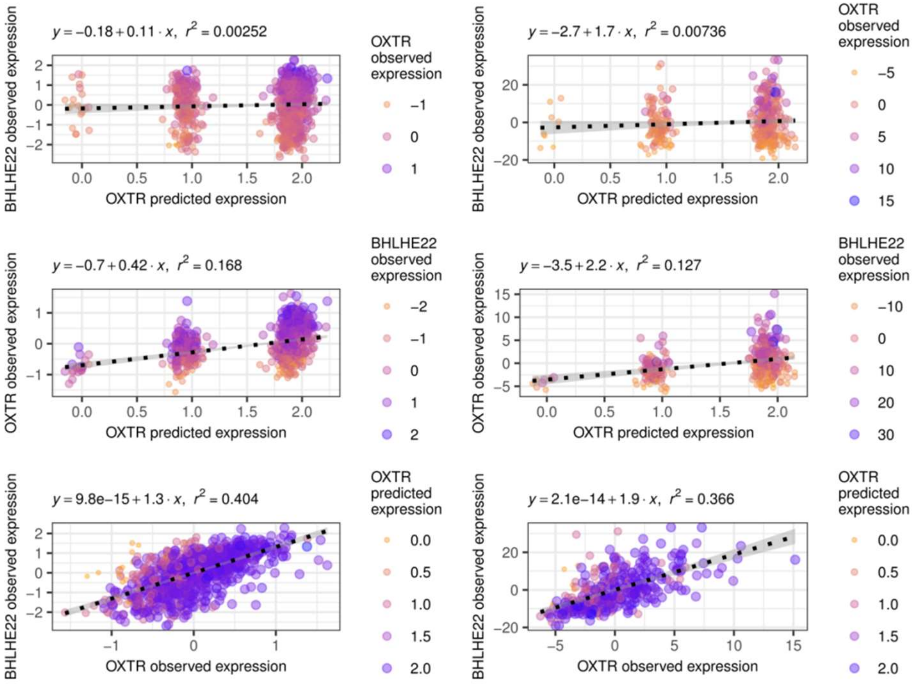

b

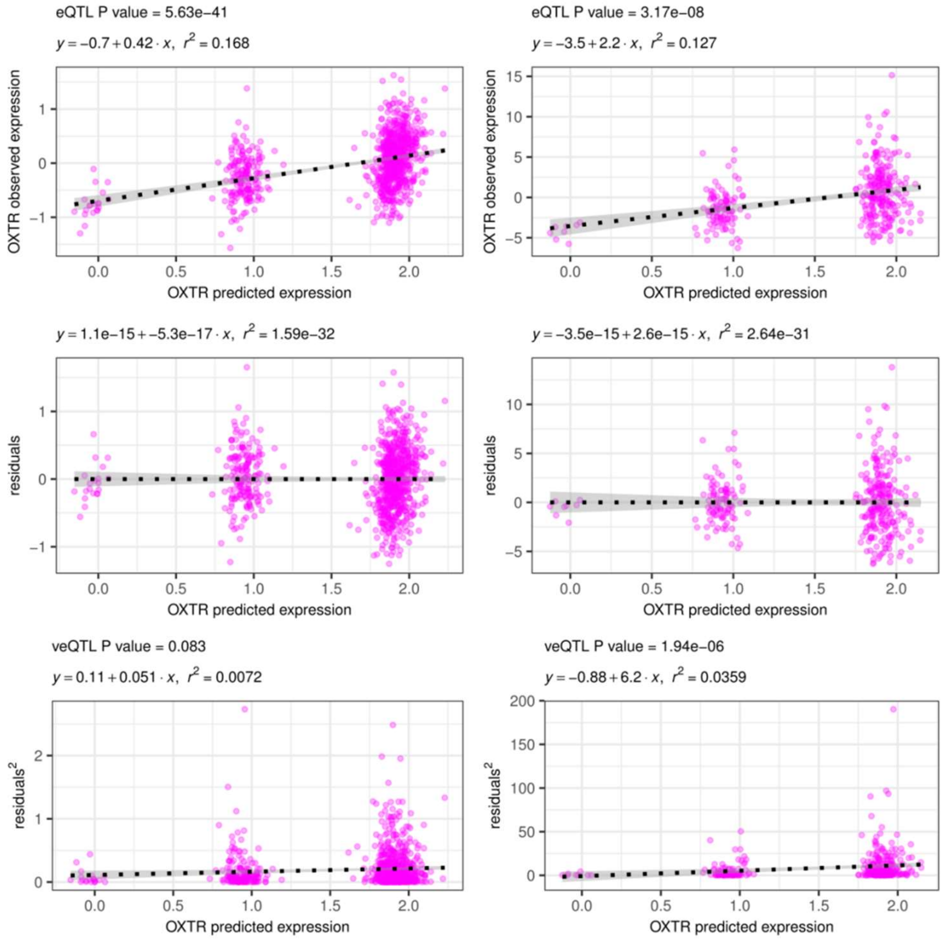

Supplementary Figure 5. Interaction plots for *OXTR* with *BHLHE22.*

a Across both datasets *OXTR* shows evidence of an interaction between its *cis*-regulatory haplotype and the expression of a second gene, *BHLHE22*, however evidence of haplotype-dependent variance (**b**) is not quite statistically significant in the Lothian Birth Cohort 1936 (P = 0.083) and is marginal (P = 0.0359) in the 1000 Genomes dataset. P and FDR values in (**a**) derived from F-tests, while P values in (**b**) derived from Spearman's rho test.

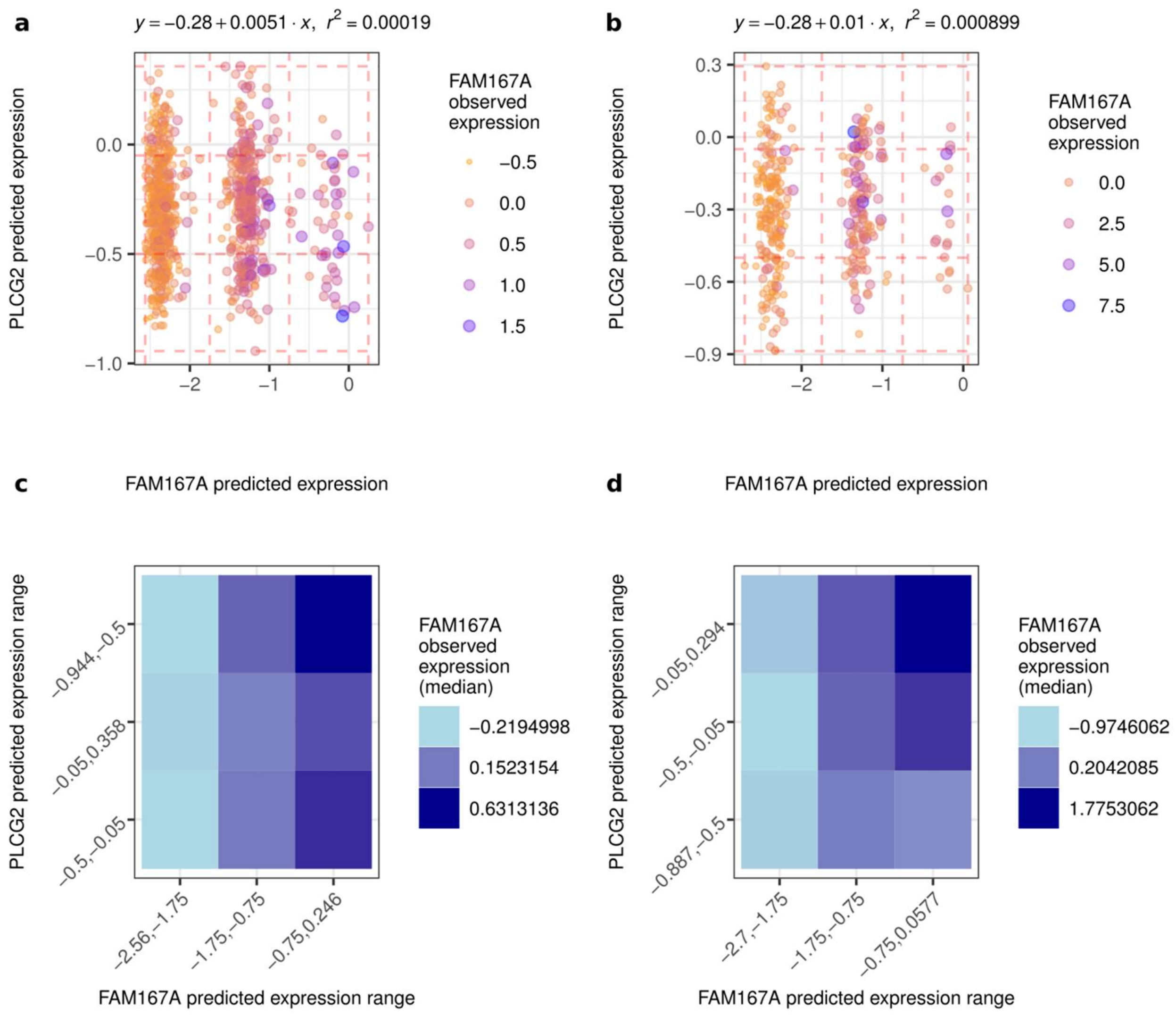

Supplementary Figure 6. Significant *cis*-genetic interaction between *FAM167A* and *PLCG2.*

FAM167A and *PLCG2* are the only gene pair that demonstrated a significant potential genetic interaction. This is shown to be the case for the Lothian Birth Cohort (**a**) and is reproduced in the 1000 Genomes data (**b**), with both datasets exhibiting statistical significance (FDR < 0.05) and sign concordance for the interaction term coefficient. The dashed grid lines mark the predicted expression ranges used to generate a simplified visualisation of the data (**c-d**), in which the median of the observed expression of *FAM167A* is presented for each of the corresponding *cis*-regulatory haplotypes (predicted expression ranges) for each gene.

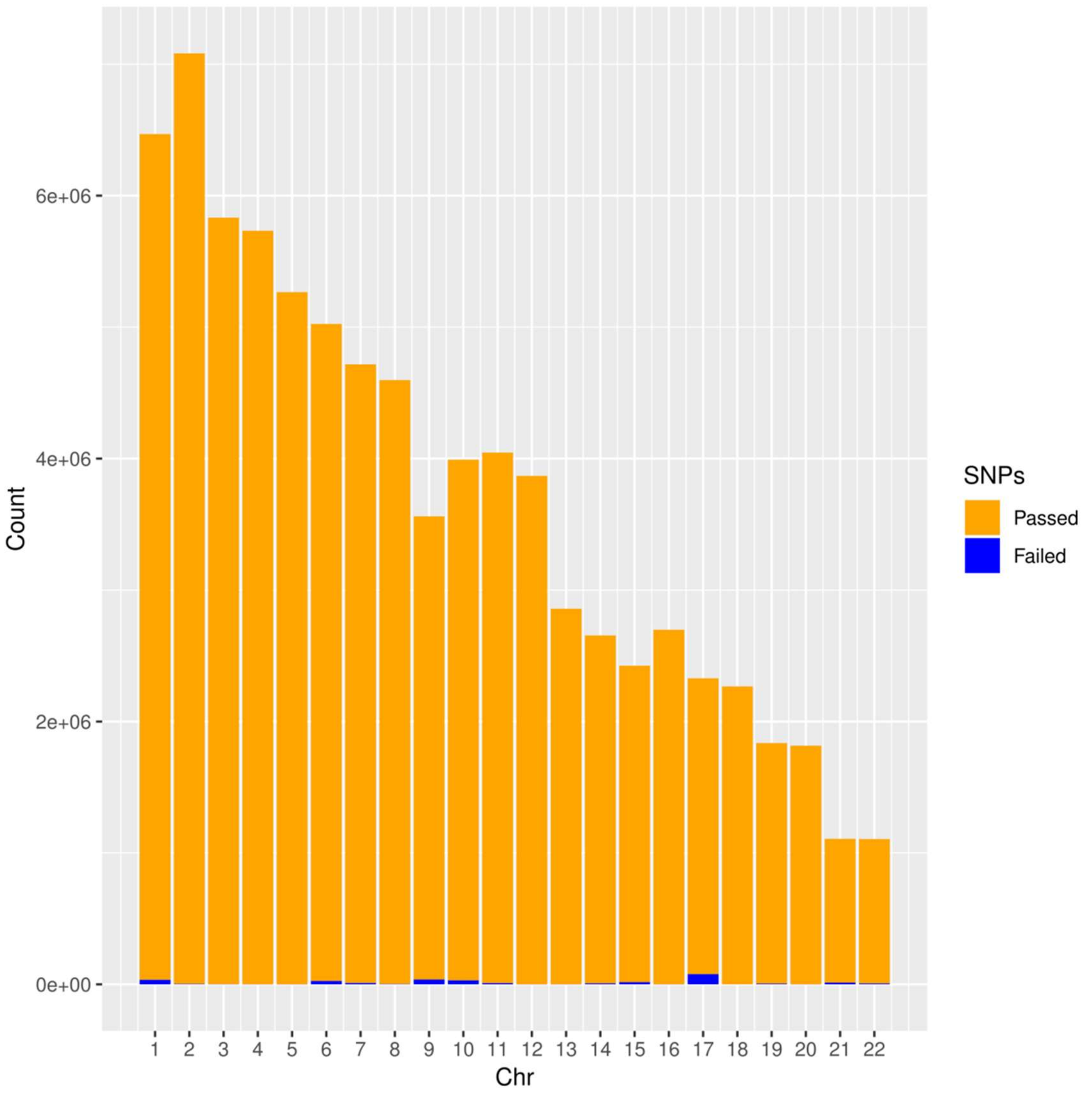

Supplementary Figure 7. Summary of genomic positions updated from GRCh37 to GRCh38 using CrossMap.

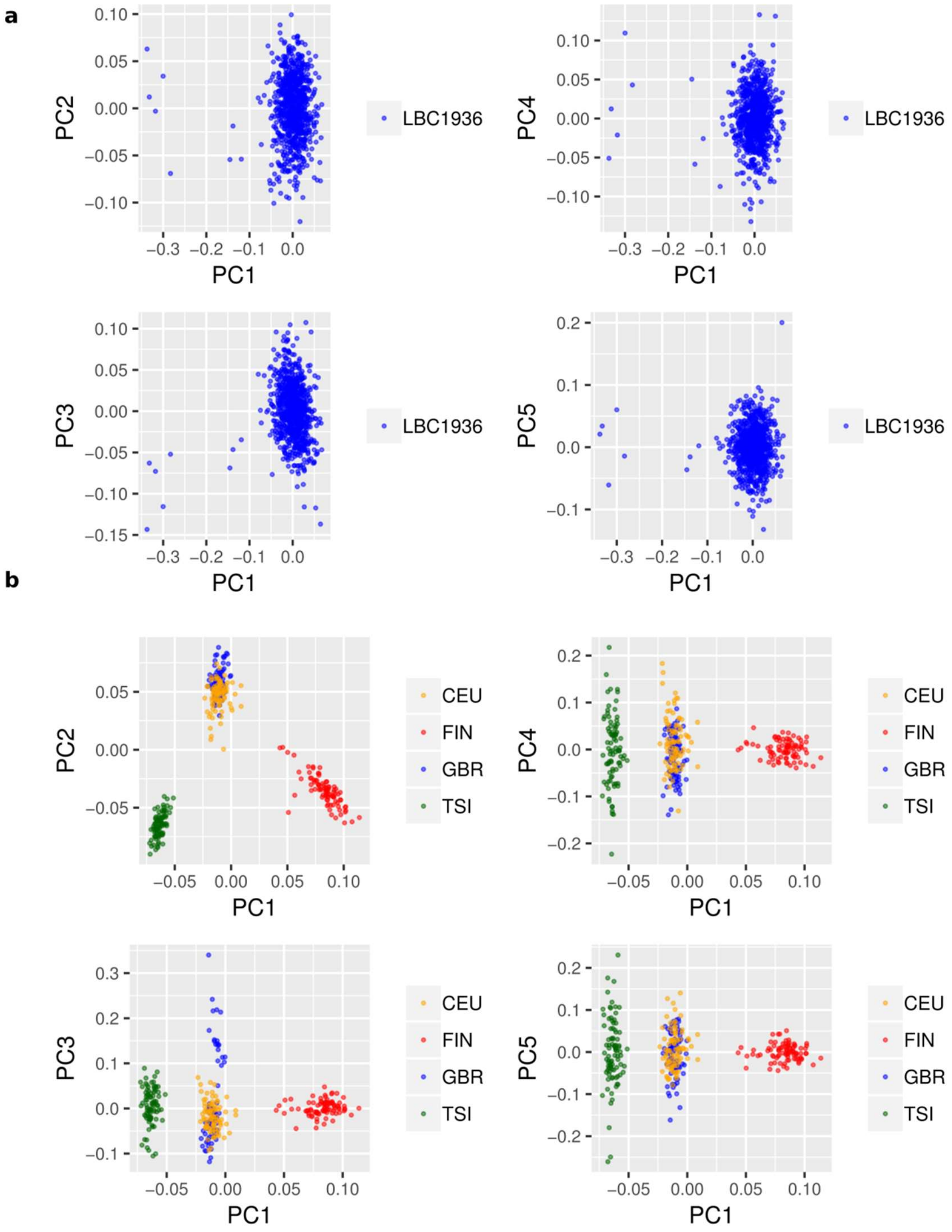

Supplementary Figure 8. Population principal components analyses

Principal components analyses (PCA) are based on SNPs exhibiting low linkage disequilibrium (LD) in the Lothian Birth Cohort 1936 (**a**) and 1000 Genomes (**b**) genotype datasets.

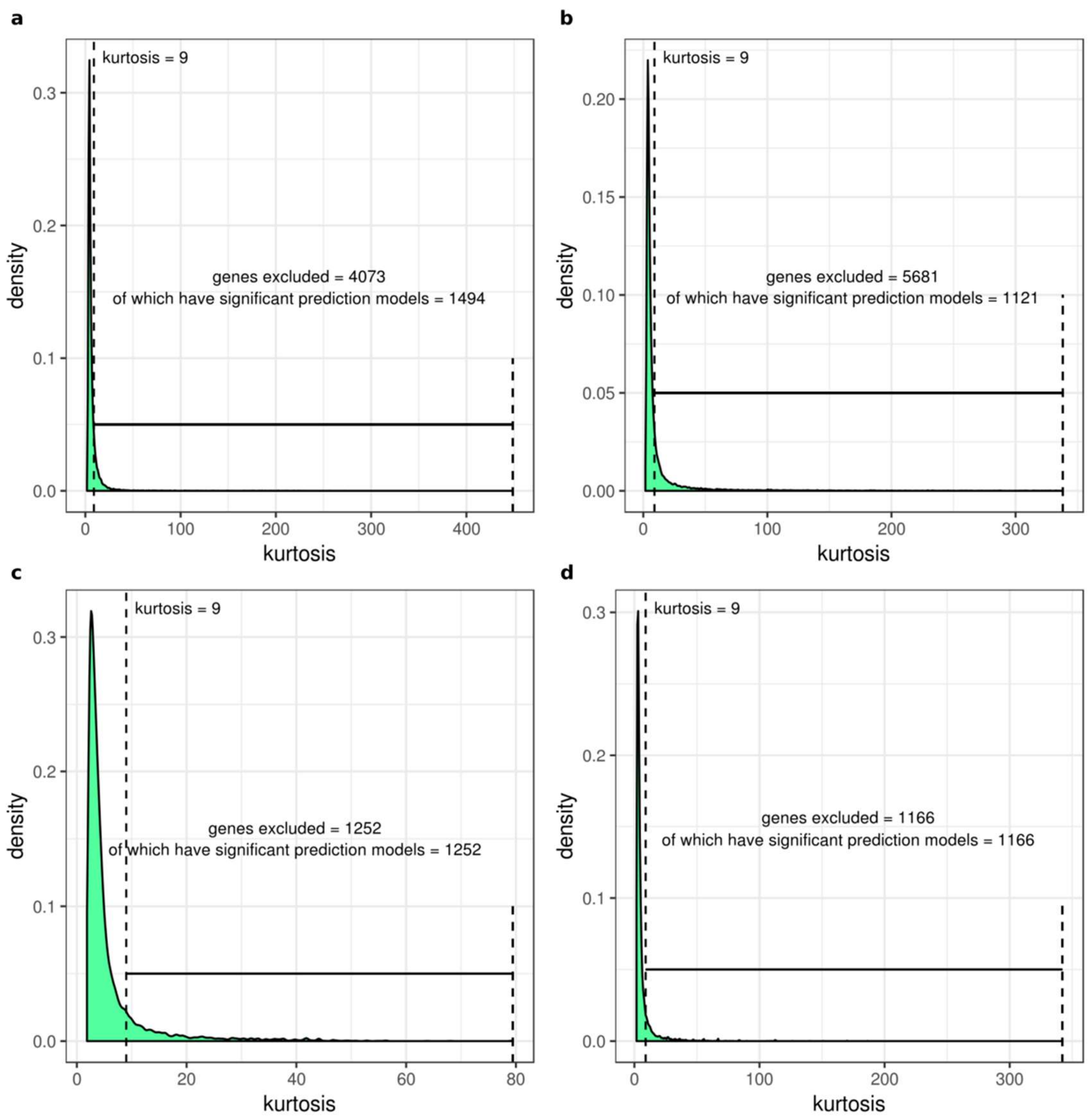

Supplementary Figure 9. Kurtosis density plots.

Kurtosis values are presented for the Lothian Birth Cohort 1936 and 1000 Genomes datasets for the observed (**a** and **b**) and predicted (**c** and **d**) gene expression data, respectively.

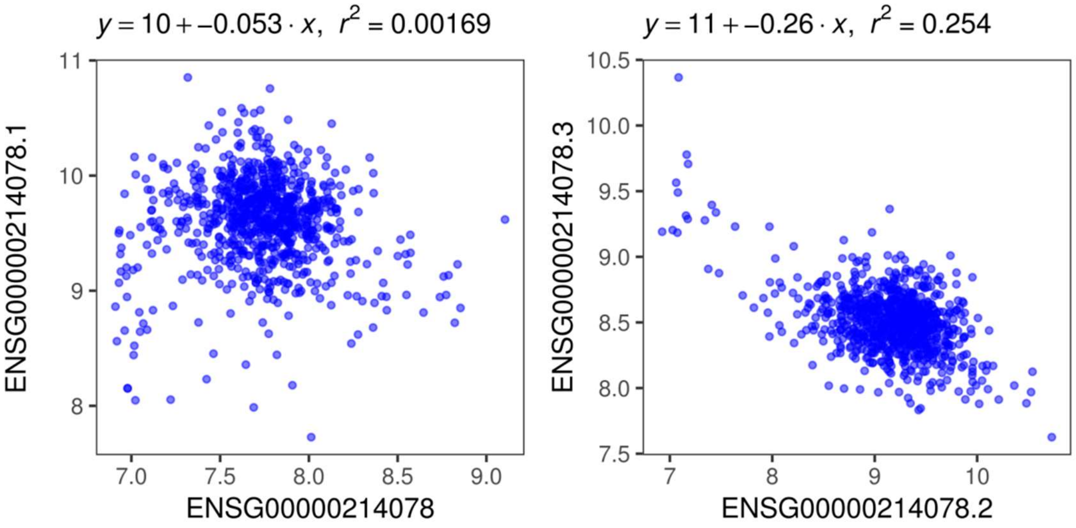

Supplementary Figure 10. Scatter plot of expression values for different probes associated with the same gene in the Lothian Birth Cohort 1936.

This figure illustrates that multiple probes for some genes are reporting the expression of different isoforms of the same gene. The application of approaches to attempt to remove the redundancy of multiple probes therefore carries the inherent risk of potentially increasing noise or missing isoform effects. In this study we have treated each probe independently.

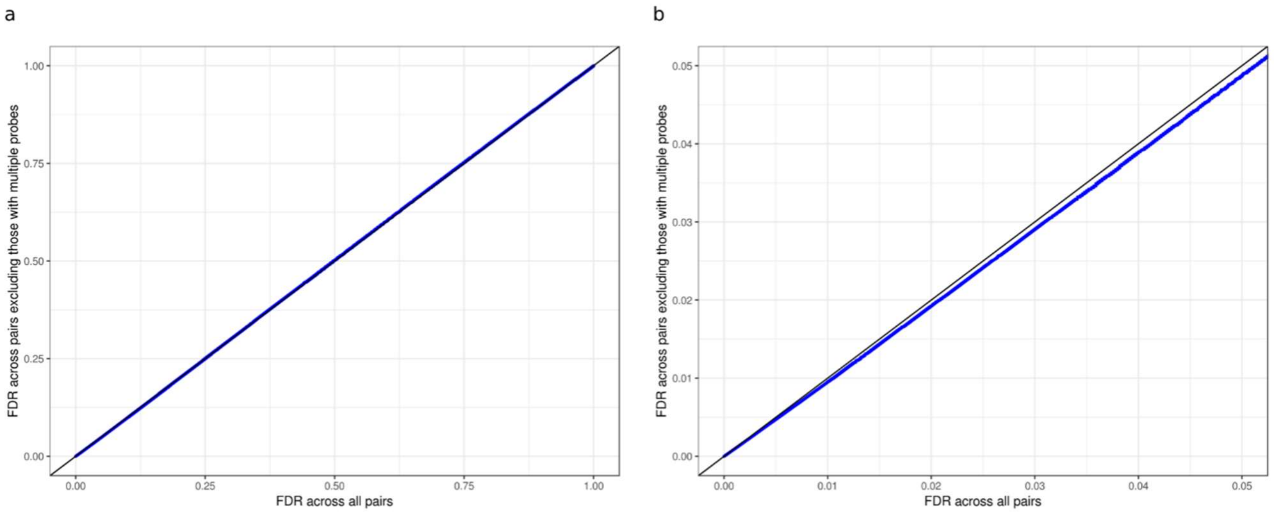

Supplementary Figure 11. F-test FDR values of single probe genes before and after excluding pairs involving multiple probes.

To test whether the presence of multiple probes was skewing the F-test P value distribution and consequently the FDR statistics, we recalculated the FDR values from Eqn 3 in the main text having excluded genes with multiple probes. As illustrated (**a**), the FDR values of the remaining genes were almost perfectly correlated to those when calculated including multiple-probe genes, suggesting that the presence of multiple probes for genes was not substantially skewing this analysis. Closer inspection of FDR values < 0.05 (**b**) illustrates deviation from the diagonal (intercept = 0, slope = 1), demonstrating that the values, although very highly correlated, are not identical.
