# Supplementary material for: Using regulatory variants to detect gene–gene interactions identifies networks of genes linked to cell immortalisation
Source: Nat Commun. 2020 Jan 17;11:343. doi: 10.1038/s41467-019-13762-6 (PMC6969137; doi:10.1038/s41467-019-13762-6)
Supplement: Supplementary file 3 — Description of Additional Supplementary Files [file 41467_2019_13762_MOESM3_ESM.pdf]

Description of Additional Supplementary Files

File Name: Supplementary Data 1
Description: Variance eQTL values for genes with reproducible cis-regulatory expression.

File Name: Supplementary Data 2
Description: Significant gene-gene interactions in LBC1936 and GEUVADIS datasets.

File Name: Supplementary Data 3
Description: Significance of gene-gene interactions in LBC1936 dataset after accounting for interacting SNPs.

File Name: Supplementary Data 4
Description: Significance of gene-gene interactions in GEUVADIS dataset after accounting for interacting SNPs.

File Name: Supplementary Data 5
Description: Significant gene-gene interactions that can be explained in LBC1936 dataset by an interacting gene.

File Name: Supplementary Data 6
Description: Significant gene-gene interactions that can be explained in GEUVADIS dataset by an interacting gene.

File Name: Supplementary Data 7
Description: Correlation of gene co-expression between GEUVADIS and LBC1936 datasets.

File Name: Supplementary Data 8
Description: Significance of gene-gene interactions when considering only cis-regulatory variants of both genes.

File Name: Supplementary Data 9
Description: Significance of gene-gene interactions when accounting for cis-regulatory variants proximal to geneB.

File Name: Supplementary Data 10
Description: Output of gene enrichment analysis across the largest networks with FUMA.

File Name: Supplementary Data 11
Description: Variance explained by genetic interactions.

File Name: Supplementary Data 12
Description: Stepwise regression over 100 iterations to identify interacting predictors that best explain variance.

File Name: Supplementary Data 13

Description: Output of FastQTL using LBC1936 dataset.

File Name: Supplementary Data 14
Description: Output of FastQTL using GEUVADIS dataset.

File Name: Supplementary Data 15
Description: Significant gene-gene interactions with concordant coefficient signs in LBC1936 and GEUVADIS datasets based on single best eQTL identified by FastQTL.
