# Supplementary material for: Using regulatory variants to detect gene–gene interactions identifies networks of genes linked to cell immortalisation
Source: Nat Commun. 2020 Jan 17;11:343. doi: 10.1038/s41467-019-13762-6 (PMC6969137; doi:10.1038/s41467-019-13762-6)
Supplement: Supplementary file 19 — Reporting Summary [file 41467_2019_13762_MOESM19_ESM.pdf]

nature research

Corresponding author(s): James Prendergast

Last updated by author(s): Sep 26, 2019

Reporting Summary

Nature Research wishes to improve the reproducibility of the work that we publish. This form provides structure for consistency and transparency in reporting. For further information on Nature Research policies, see Authors & Referees and the Editorial Policy Checklist .

Statistics

For all statistical analyses, confirm that the following items are present in the figure legend, table legend, main text, or Methods section.

n/a	Confirmed	
☐	☒	The exact sample size (*n*) for each experimental group/condition, given as a discrete number and unit of measurement
☒	☐	A statement on whether measurements were taken from distinct samples or whether the same sample was measured repeatedly
☐	☒	The statistical test(s) used AND whether they are one- or two-sided<br>*Only common tests should be described solely by name; describe more complex techniques in the Methods section.*
☐	☒	A description of all covariates tested
☐	☒	A description of any assumptions or corrections, such as tests of normality and adjustment for multiple comparisons
☐	☒	A full description of the statistical parameters including central tendency (e.g. means) or other basic estimates (e.g. regression coefficient) AND variation (e.g. standard deviation) or associated estimates of uncertainty (e.g. confidence intervals)
☐	☒	For null hypothesis testing, the test statistic (e.g. *F*, *t*, *r*) with confidence intervals, effect sizes, degrees of freedom and *P* value noted<br>*Give P values as exact values whenever suitable.*
☒	☐	For Bayesian analysis, information on the choice of priors and Markov chain Monte Carlo settings
☒	☐	For hierarchical and complex designs, identification of the appropriate level for tests and full reporting of outcomes
☒	☐	Estimates of effect sizes (e.g. Cohen's *d*, Pearson's *r*), indicating how they were calculated

Our web collection on statistics for biologists contains articles on many of the points above.

Software and code

Policy information about availability of computer code

Data collection	No software was used
Data analysis	GATK v3.5-0-g36282e4 (GenotypeGVCFs, CombineGVCFs, ApplyRecalibration, SelectVariants), GATK v3.4-0-g7e26428 (HaplotypeCaller) bcftools v1.3.1, htslib v1.3.1, Plink v1.90b4, vcftools v0.1.13, R v3.3.3 and v3.5.1, tabix v0.2.5, PredictDB, PrediXcan, PEER v1.3, FUMA.

For manuscripts utilizing custom algorithms or software that are central to the research but not yet described in published literature, software must be made available to editors/reviewers. We strongly encourage code deposition in a community repository (e.g. GitHub). See the Nature Research guidelines for submitting code & software for further information.

Data

Policy information about availability of data

All manuscripts must include a data availability statement. This statement should provide the following information, where applicable:

- Accession codes, unique identifiers, or web links for publicly available datasets
- A list of figures that have associated raw data
- A description of any restrictions on data availability

Genotype and normalised gene expression data for the GEUVADIS dataset is available at: ftp.1000genomes.ebi.ac.uk/vol1/ftp/release/20130502 and https://www.ebi.ac.uk/arrayexpress/files/E-GEUV-1/analysis_results/, respectively. Sequence data for the Lothian Birth Cohort has been deposited at the European Genome-phenome Archive (EGA), which is hosted by the EBI and the CRG, under accession numbers EGAS00001003818 and EGAS00001003819.

Field-specific reporting

Please select the one below that is the best fit for your research. If you are not sure, read the appropriate sections before making your selection.

☒ Life sciences ☐ Behavioural & social sciences ☐ Ecological, evolutionary & environmental sciences

For a reference copy of the document with all sections, see nature.com/documents/nr-reporting-summary-flat.pdf

Life sciences study design

All studies must disclose on these points even when the disclosure is negative.

Sample size	Data for 2 independent populations of 930 and 358 individuals, respectively were analysed. These samples were chosen because they were both of European ancestry and had complementary genotype and gene expression data.
Data exclusions	Genotype data was filtered to remove individuals exhibiting high relatedness, and SNPs filtered to remove those in high linkage disequilibrium. These are standard pre-established filtering steps to address issues arising from population structure.
Replication	The study was designed to test for replication of the findings in one population using an independent population. It also benefits from the gene expression data being generated using different technologies, which addresses the likelihood of any results being due to normalisation effects. Replication was confirmed for a large number of the gene-gene interactions identified.
Randomization	Random assignment of individuals to groups is not relevant in this study. Covariates were controlled by addressing population structure, applying a kurtosis filter to observed gene expression, PEER normalisation, and the inclusion of covariates in the statistical models.
Blinding	Blinding is not relevant to this study. All datasets were generated prior to the conception of this study and the populations analysed are independent of one-another.

Reporting for specific materials, systems and methods

We require information from authors about some types of materials, experimental systems and methods used in many studies. Here, indicate whether each material, system or method listed is relevant to your study. If you are not sure if a list item applies to your research, read the appropriate section before selecting a response.

Materials & experimental systems

n/a	Involved in the study
☒	☐ Antibodies
☐	☒ Eukaryotic cell lines
☒	☐ Palaeontology
☒	☐ Animals and other organisms
☐	☒ Human research participants
☒	☐ Clinical data

Methods

n/a	Involved in the study
☒	☐ ChIP-seq
☒	☐ Flow cytometry
☒	☐ MRI-based neuroimaging

Eukaryotic cell lines

Policy information about cell lines

Cell line source(s)	Gene expression data was obtained from human lymphoblastoid cell lines
Authentication	NA/Not authenticated
Mycoplasma contamination	Not tested
Commonly misidentified lines (See ICLAC register)	*Name any commonly misidentified cell lines used in the study and provide a rationale for their use.*

Human research participants

Policy information about studies involving human research participants

Population characteristics	The cohort used was the Lothian Birth Cohort (https://www.lothianbirthcohort.ed.ac.uk/content/scottish-mental-survey-1947) consisting of elderly individuals, born within a year and of mixed gender
Recruitment	The participants were originally recruited as had participated in the same mental health survey at school for more details see https://www.lothianbirthcohort.ed.ac.uk/content/scottish-mental-survey-1947
Ethics oversight	Ethics permission was obtained from the Multi-Centre Research Ethics Committee for Scotland (Wave 1: MREC/01/0/56), the Lothian Research Ethics Committee (Wave 1: LREC/2003/2/29), and the Scotland A Research Ethics Committee (Wave 2: 07/MRE00/58). All persons gave their informed consent prior to their inclusion in the study.

Note that full information on the approval of the study protocol must also be provided in the manuscript.
